# Detection of polyphenols-to-amino acids ratio in Wuyi Rock Tea *via* CARS-PLSR processing of near infrared spectroscopy

**DOI:** 10.1039/d5ra08177g

**Published:** 2026-01-08

**Authors:** Zhenyi Xu, Xianbiao Jiang, Qingqing Chen, Pumo Cai

**Affiliations:** a College of Tea and Food Science, Wuyi University Wuyishan 354300 People's Republic of China caipumo@wuyiu.edu.cn +86-599-5137553

## Abstract

The total polyphenols (TP), free amino acids (FAA), and the polyphenols-to-amino acids ratio (TP/FAA) serve as crucial indicators of the taste quality of tea. Traditional detection methods, however, often suffer from limitations such as prolonged analysis time, complex procedures, and the potential for reagent contamination. To expedite the determination and analysis of the TP/FAA in Wuyi Rock Tea, this study employed three wavelength selection methods: Uninformative Variable Elimination (UVE), Successive Projections Algorithm (SPA), and Competitive Adaptive Reweighted Sampling (CARS). These approaches were integrated with Partial Least Squares Regression (PLSR) and Principal Component Regression (PCR) to develop a quantitative analysis model for the polyphenols-to-amino acids ratio in Wuyi Rock Tea. Results revealed that after applying Standard Normal Variate (SNV) combined with Savitzky–Golay Derivative (SD) preprocessing to the near-infrared (NIR) spectra, all three methods improved model performance to varying extents, with the CARS-PLSR wavelength selection method demonstrating the most significant optimization. The coefficients of determination for both calibration and prediction sets of the TP/FAA ratio reached 0.9897 and 0.9812, respectively, while the root mean square error of calibration (RMSEC) and prediction (RMSEP) were 0.1854 and 0.1434, respectively. The relative percent deviation (RPD) was 3.21, indicating enhanced stability and accuracy of the quantitative model. Validation results confirmed that the CARS-PLSR method effectively extracted essential NIR spectral variables while concurrently eliminating redundant spectral noise. This study presents a novel framework for rapid tea quality assessment using NIR spectroscopy.

## Abstract 2

Based on near-infrared spectroscopy and the CARS-PLSR algorithm, rapid detection of the TP/FFA in Wuyi Rock Tea is achieved.

## Graphical abstract

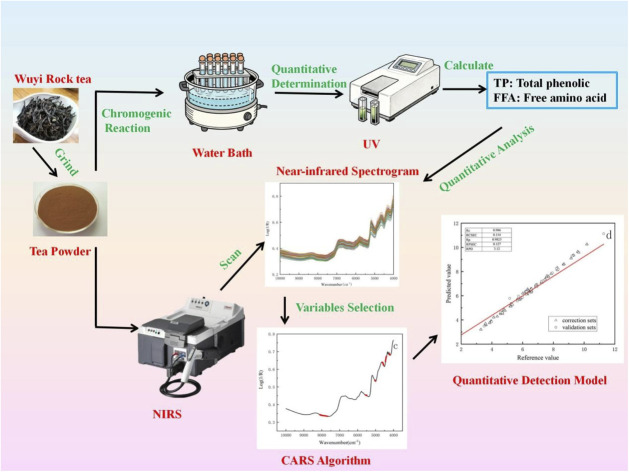

## 1. Introduction

Wuyi Rock Tea is widely regarded as a special grade of oolong teas due to its exceptional quality characteristics, which are primarily assessed through aroma and taste.^1^ The global reputation of Wuyi Rock Tea is deeply rooted in its biochemical composition, particularly the concentrations of key components such as tea polyphenols, amino acids, caffeine, and tea polysaccharides. The total polyphenols content (TP) significantly contributes to the tea's mellow taste, with the astringency largely attributed to catechins, a major component of TP, especially esterified catechins such as epigallocatechin gallate (EGCG).^2,3^ Concurrently, the total amount of free amino acids (FAA) determines the freshness of the tea infusion,^4^ especially the presence of theanine. Theanine can mitigate the astringency caused by EGCG while caffeine enhances the sweetness associated with theanine.^5^ Therefore, the balance between astringency and freshness is crucial for the flavor profile of tea, with the ratio of total polyphenols to free amino acids (TP/FAA) serving as an important indicator of tea flavor quality,^6,7^ demonstrating that the TP/FAA ratio provides a more accurate representation of tea flavor than relying solely on individual indices of amino acid or tea polyphenol content. Furthermore, this ratio exhibited a significant negative correlation with tea qualities such as freshness, body and overall flavor profiling. Similarly, Guo *et al.*^8^ confirmed that the TP/FAA ratio was an important metric for assessing the balance between flavor mellowness and astringency, thereby serving as a critical indicator for evaluating tea processing suitability.

In the analysis of tea, the detection of TP and FAA content is typically conducted following established standard methods, including ISO Standards (ISO 14502-1:2005, ISO 19563-2017) and Chinese national standard (GB/T8313-2008, GB/T8314-2013). Common analytical techniques employed in these methods include High-Performance Liquid Chromatography (HPLC),^8^ Ultraviolet detection (UV),^9^ Ultra-High-Performance Liquid Chromatography-Tandem Mass Spectrometry (UHPLC-MS/MS),^10^ Ultra-Fast Liquid Chromatography Quadrupole Time of Flight Tandem Mass Spectrometry (UFLCQ-TOF-MS/MS),^11^ and Gas Chromatograph-Mass Spectrometer (GC-MS).^12^ These methods are known for their high accuracy and sensitivity in detecting chemical constituents. However, they are often characterized by time-consuming, labor-intensive, and resource-demanding, including costly instrumentation and maintenance. Such as the complex tea matrix (pigments, polysaccharides) can easily lead to column efficiency degradation, requiring frequent maintenance.^13^ Additionally, solid-phase extraction(SPE) or repeated organic solvent extraction was often necessary, which may affect recovery rates.^14^ Furthermore, low ionization efficiency in mass spectrometry analysis necessitates isotope internal standard calibration.^15^ Therefore, these conventional techniques may not be well-suited for rapid routine quality screening of finished teas or real-time quality monitoring in practical production scenarios.

The near-infrared spectroscopy (NIR) was a form of electromagnetic radiation that exists between visible light and mid-infrared radiation. It mainly reflects the combined and double harmonic absorption spectrum information from the vibrational modes of hydrogen-containing groups, such as C–H, N–H, and O–H in organic compounds. Due to its advantages including low cost, high efficiency, rapid response time, eco-friendliness, and non-destructive nature, NIR technology has gained widespread application in tea quality assessment.^16–18^ However, the data generated from near-infrared spectroscopy is voluminous and often large contains not only useful information pertinent to the analytes but also a significant amount of redundant data. This excess information can detrimentally affect the accuracy of both quantitative and qualitative analyses. As a result, variable selection methods are frequently employed to extract effective spectral characteristic wavelengths for accurate analysis. For instance, Chen *et al.*^19^ used principal component analysis (PCA) to extract low-dimensional features from the near-infrared spectrum and subsequently employed a backpropagation adaptive lifting algorithm to establish predictive models related to the sensory characteristics of eight different types of tea. Similarly, Dong *et al.*^20^ used the competitive adaptive reweighted sampling (CARS) technique to identify optimal near-infrared spectral intervals for a Si-PLS model, integrating it with an extreme learning machine and adaptive promotion algorithm to facilitate rapid detection of theaflavins to aerythromycin ratios during the fermentation process of Gongfu black tea, achieving a coefficient of determination (*R*^2^) of 0.893 and a root mean square error of prediction(RMSEP) of 0.0044. Moreover, Tan *et al.*^18^ used partial least squares discriminant analysis(PLS-DA) in conjunction with near-infrared spectroscopy to classify six distinct types of tea, including Biluochun, Xihu Longjing, Xinyang Maojian, Qihong, Tieguanyin and Yinzhen. Meanwhile,^21^ integrated Fourier transform near-infrared spectroscopy with multivariate statistical techniques to develop a partial least squares(PLS) model for the rapid determination of ethyl valeric ester in liquor, demonstrating a central determination coefficient of 0.964 and an RMSEP of 0.023.

Although these studies have demonstrated the potential of near-infrared technology in tea analysis, most of the research has focused on general quality parameters or specific compound categories, and these studies have mostly been conducted for a wide range of tea types. To date, there has been no dedicated study that systematically optimizes both spectral preprocessing and wavelength selection for the prediction of the TP/FAA ratio in Wuyi Rock Tea. Unlike other types of tea, Wuyi Rock Tea has unique processing methods and complex procedures (including picking, withering, fermentation, shaping, rolling, drying, sorting, baking, grading, and blending),^22,23^ which leads to a rich content of its biochemical components and poses challenges for establishing a rapid near-infrared detection method. Conventional NIR models often struggle to accommodate this complexity due to insufficient optimization of spectral preprocessing steps, which can lead to residual noise and baseline drift.^24^ Therefore, a near-infrared analysis method specifically tailored for rapid detection of the ammonia-phenol ratio in Wuyi Rock Tea needs to be developed. This method should combine advanced preprocessing with intelligent variable selection to improve the prediction accuracy and reliability for this specific application.

To address these gaps, this study aimed to improve the prediction efficiency of the TP/FAA ratio in Wuyi Rock Tea and enhance model performance. A series of mathematical signal preprocessing techniques, including Multiplicative Scatter Correction(MSC), Standard Normal Variable(SNV), Sawiecki–Golay filter(SG), first derivative(FD) and second derivative(SD), were employed to preprocess the spectra data. Subsequently, Competitive Adaptive Reweighted Sampling(CARS), Successive Projections Algorithm(SPA), and Uninformative Variable Elimination(UVE) were used to optimize the original spectra data. Finally, models for predicting the TP/FAA ratio in Wuyi Rock Tea were established using Partial Least Squares Regression(PLSR) or Principal Component Regression(PCR). The novelty of this work lies in the systematic integration of preprocessing optimization with advanced wavelength selection specifically tailored for the accurate and rapid non-destructive assessment of the TP/FAA balance in Wuyi Rock Tea, offering a robust tool for quality control and process monitoring.

## 2. Materials and methods

### 2.1 Sample preparation and instrumentation

A total of 99 samples of Wuyi Rock Tea, sourced from various production areas and manufacturers in Wuyishan City, were acquired for this study. The samples were ground into a fine powder using a small electric mill and subsequently passed through a 50-mesh sieve to ensure uniform particle size. Given the well-documented and substantial influence of moisture content on NIR spectral profiles, stringent measures were taken to minimize its variability. All sample preparation and spectral acquisition procedures were performed in a climate-controlled laboratory maintained at 25 ± 1 °C with relative humidity below 60%. Immediately following the grinding and sieving process, the tea powder was transferred into resealable, moisture-barrier bags to prevent hygroscopic absorption from the atmosphere. These sealed samples were then stored in a desiccator containing silica gel until the moment of NIR measurement. This protocol aimed to stabilize the physical state of the samples and reduce moisture-induced spectral baseline drift and scattering effects, thereby isolating spectral variance more closely related to the target chemical constituents (TP and FAA).

The spectra were collected using the Antaris II near-infrared spectrometer (Thermo Fisher Scientific Company, USA) in diffuse reflection mode with an integrating sphere. The spectral acquisition parameters were listed as follows: the wavelength range was 4000–10 000 cm^−1^, with a resolution of 4 cm^−1^, a total of 64 scans, and the acquisition mode utilizes integrating sphere diffuse reflection. Spectral preprocessing and data optimization analysis were performed by TQ Analyst, PyCharm and Origin software.

### 2.2 Determination of TP/FAA ratios

Total phenolic (TP) content was determined in accordance with the international standard method (ISO 14502-1-2005). Free amino acid(FAA) content was assessed following the guidelines outlined in ISO19563-2017. To evaluate the product quality of the tea, the ratio of TP/FAA was calculated by dividing the total phenolic content by the free amino acid content.

### 2.3 NIR spectra acquisition and preprocessing

After allowing the NIR spectrometer to preheat, the NIR spectrum acquisition mode is configured. The sampling method employed was integrating sphere diffuse reflection, covering a spectral scanning range of 10 000 to 4000 cm^−1^, with a resolution of 8 cm^−1^. Each sample underwent 64 scans, resulting in a total of 7469 spectral data points per measurement. The spectra was recorded in absorbance format. Each sample was measured in duplicate, and the average of the two spectra measurements was used as the final spectral data of that sample.

The raw NIR spectral data obtained from the tea samples contained background and external noise, primarily arising from variations in transmitted and scatter light due to heterogeneous particle size of the tea samples.^24,25^ To reduce the effects of particle dispersion, surface variability, and background noise, various spectral preprocessing techniques were applied. Commonly used preprocessing methods included Multiplicative Scattering Correction(MSC), Standard Normal Variate transformation (SNV), Savitzky–Golay filter(SG), first-order derivative(FD), and second-order derivative(SD).^26^ The different spectral preprocessing methods and their respective effects were detailed in Table 1. Before modeling the original spectral data, we applied both independent and combined preprocessing methods to ensure the robustness and accuracy of the calibration algorithms.

**Table 1:** The different spectral pretreatment methods and their effects

Objective	Spectral preprocessing method	Effect
Baseline correction	FD	Improve spectral resolution and eliminate instrument background interference
	SD	
Scattering correction	SNV	Eliminate the scattering of light source caused by uneven particle size and distribution
	MSC	
Smoothing	SG	Noise cancellation

### 2.4. Modeling methods

#### 2.4.1 Partial least squares regression (PLS) model

The Partial Least Squares (PLS) model is a classical regression approach mainly used to establish correlation between two sets of variables (*X* and *Y* variables).^27^ This approach not only addresses the issue of multicollinearity but also emphasizes the role of independent variables in interpreting and predicting dependent variables during feature vectors selection. By doing so, it eliminates the influence of unwanted noise on regression process, resulting in a model that incorporates the fewest possible variables. Given that this modelling approach deals with a substantial number of spectral variables across the full spectrum, it often encounters irrelevant and highly correlated independent variables, which can compromise the model's stability and efficiency.^28^

#### 2.4.2 Principal component regression (PCR) model

The Principal Component Regression (PCR) model aims to extract a limited number of principal components as new independent variables through principal component analysis of the original independent variables. This process allows for regression analysis with the dependent variables, effectively eliminating multicollinearity among the independent variables and simplifying the overall model structure. Essentially, PCR is a statistical method that uses mathematical dimensionality reduction to transform correlated indicators into a new set of uncorrelated composite indicators. In this approach, the independent variable *X* (spectral data) is initially analyzed through principal component analysis, and then the original independent variable, along with the dependent variable *Y* (measured value), is substituted with derived principal component factors for conducting multiple linear regression analysis.

### 2.5 Multivariate calibration analysis

#### 2.5.1 Successive projection algorithm (SPA)

SPA is a forward variable selection algorithm designed to minimize collinearity within a vector space. It begins with the first wavelength and, in each iteration, adds a new wavelength until a predetermined number of wavelengths is selected. The primary characteristic of this algorithm is its ability to choose a combination of wavelengths that contains the least amount of redundant information, effectively addressing the collinearity issue. The key advantage of SPA lies in its capacity to extract several significant feature wavelengths from the entire spectral range, thereby eliminating redundant information from the original spectral matrix. This makes it a valuable tool for screening and selecting spectral feature wavelengths.^29^

#### 2.5.2 Uninformative variable elimination (UVE)

UVE is a wavelength selection algorithm based on PLSR coefficient, which takes the regression coefficient as the most important measure of wavelength selection, and removes the spectral data containing invalid information.^30^ As a variable screening method, the characteristic wavelength is extracted as the input variable of PLSR model, so as to improve the operation speed of PLSR and improve the quantitative model prediction ability of PLSR.^31^

#### 2.5.3 Competitive adaptive reweighted sampling(CARS)

CARS is a feature variable selection method combining Monte Carlo sampling with PLS model regression coefficient, similar to the idea of genetic algorithm, which gradually evaluates, analyzes, screens and eliminates each wavelength point in the spectrum, and is suitable for high-dimensional spectral screening.^32,33^ Its can not only effectively remove non-information variables, but also reduce the influence of collinear variables on the model as much as possible, and finally select the most critical variables for the prediction target.

### 2.6 Model calibration and validation

A total of 90 samples of Wuyi Rock Tea were randomly divided into two subsets: the calibration set and the validation set. The calibration set accounts for 80% of the total sample size was used for building and optimizing the analytical model, while the validation set constituted the remaining 20% and was used to evaluate the predictive capacity of the model. To evaluate the effectiveness of the model, several statistical indicators were computed, including the correlation coefficient of the calibration set (*R*_c_), the root mean square error of calibration (RMSEC), the correlation coefficient of validation set (*R*_p_), and the root mean square error of prediction (RMSEP). A higher correlation coefficient combined with a lower root mean square error signifies better model performance. Additionally, the residual prediction deviation (RPD), which is derived from the ratio of *R*_p_ to RMSEP, serves as a crucial indicator of the reliability of the quantitative model.^31^ An RPD value greater than 3.0 indicates reliable model performance with adequate predictive accuracy suitable for practical applications; an RPD ranging from 2.5 to 3.0 suggests that the model's reliability requires enhancement, limiting its utility to mere estimation; an RPD between 2.0 and 2.5 implies approximate quantitative predictive capability; while RPD below 2.0 denotes unreliable prediction.^34^

Furthermore, nine additional samples of Wuyi Rock Tea were subjected to pretreatment. The TP and FAA contents were determined according to mentioned above, from which the TP/FAA ratio was subsequently calculated. NIR spectra for these samples were acquired using the method mentioned in part 1.3, enabling rapid determination of TP/FAA ratios through the established quantitative model. A comparative analysis between the measured and predicted datasets was conducted to validate the accuracy and stability of the quantitative analytical model. Each sample was measured in triplicate, with the mean values used for further analysis.

## 3. Results and discussion

### 3.1 TP/FAA ratio data in all samples

The ratio of TP/FAA was calculated by dividing the total amount of tea polyphenols by the total amount of amino acids. The TP/FAA ratio among the 90 Wuyi Rock Tea samples were shown in Table 2, of which 70 were selected as calibration sets and 30 as the validation sets for model development. Results showed that the ratio of phenol to ammonia in calibration set exhibited a range from 3.28 to 10.67, while the validation set demonstrated a slightly broader range of 3.49 to 11.27. These findings indicate a variable distribution of the TP/FAA ratio within the examined Wuyi Rock Tea samples, providing a robust foundation for subsequent modeling efforts.

**Table 2:** Polyphenols-to-amino acids ratio in 90 samples of Wuyi Rock Tea

Test items	Type of sample	Range of test results/%	Mean value/%
TP/FAA	Calibration sets	3.28–10.67	5.81
	Validation sets	3.49–11.27	5.74

### 3.2 Original spectral characteristics of Wuyi Rock Tea

**Fig. 1:**
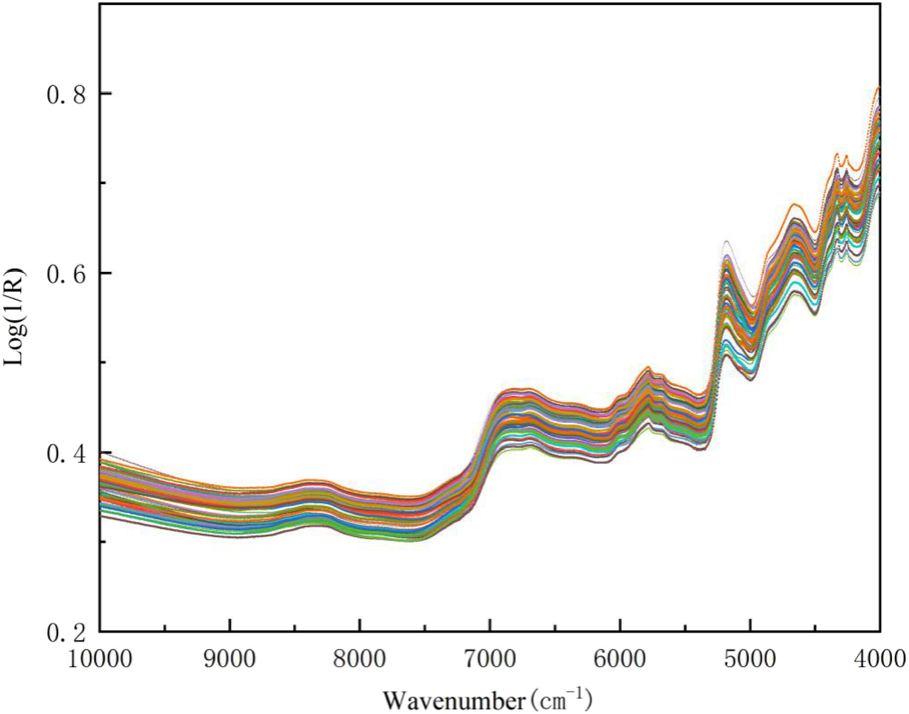
The NIR spectras of Wuyi Rock Tea(*n* = 90).

The original spectrograms of the 90 Wuyi Rock Tea samples were shown in Fig. 1, revealing a broadly similar trend among the spectral lines. Notably, the samples exhibited analogous absorption peaks, indicating that they share similar chemical compositions. The variation in peak intensity may be attributed to differences in the concentrations of specific components within the samples. The prominent absorption band observed in the range of 7000–8000 cm^−1^ corresponds to the first-order frequency-doubled combined absorption band of C–C, C–O, and C–N bonds. Additionally, the peak at 6917 cm^−1^ represents the first co-frequency combinde absorption bands of C=C, C=O, and C=N, which are mainly related to flavonoids.^35^ Furthermore, the absorption bands identified at 4000–4425 cm^−1^ and 4628–4765 cm^−1^ pertain to the combination of stretching and deformation of C–H bonds, as well as combination of stretching and bending of N–H bonds, both of which are related to tea polyphenols.^36^ In addition, it is apparent from the figure that the NIR spectra exhibit considerable backgroud interference and baseline drift. Consequently, effective spectral pretreatment is essential to eliminate these effects and enhance the reliability of subsequent analyses.

### 3.3 Optimization of spectral pretreatment method

To optimize the spectral preprocessing method and analysis model, quantitative data derived from the original NIR spectra were analyzed using various spectral signal preprocessing techniques under both PLSR and PCR methodologies. The optimization results, displayed in Table 3, indicate that the PLSR approach yielded superior model performance. This is primarily attributed to the fact that, in the PCR method, the chemical measurement matrix (*Y*) is typically examined through principal component analysis, with regression conducted based on abstract factors, while the concentration matrix (*X*) remains unprocessed. In contrast, the PLSR method simultaneously decomposed the (*X*) and (*Y*) matrix through principal component analysis, allowing for regression based on principal factor, which improved the reliability of the model.^37,38^ The PLS model demonstrates a robust capability to manage multicollinearity and high-dimensional data, effectively extracting latent variables that have greatest relevant to the response variable. This characteristic renders it particularly suitable for the quantitative analysis of NIR spectroscopy data, corroborating the findings of Herve.^39^ It is noteworthy that while the combination MSC + FD + SG yielded a higher RPD value (3.08) compared to FD + SG (2.42), the latter was selected for subsequent wavelength selection and model optimization. This decision was based on a holistic evaluation of model performance metrics. The primary objective is to obtain a model with high and consistent predictive accuracy. The model based on FD + SG preprocessing demonstrated a superior and more stable prediction coefficient for the validation set (*R*_p_ = 0.9420) compared to that based on MSC + FD + SG (*R*_p_ = 0.8844). Furthermore, the calibration and prediction coefficients for the FD + SG model were notably closer (*R*_c_ = 0.9447, *R*_p_ = 0.9420), indicating excellent model robustness and a lower likelihood of overfitting. In contrast, the larger discrepancy between *R*_c_ (0.9424) and *R*_p_ (0.8844) observed for the MSC + FD + SG model suggests potential challenges in generalization. Although RPD is a valuable comprehensive indicator, the optimization priority was placed on maximizing and stabilizing the predictive power (*R*_p_) for the target analyte. Consequently, FD + SG was identified as the most effective and reliable preprocessing method for this study.

**Table 3:** The effects of different pretreatment methods on the PLS or PCR regression models

Processing methods	Model	Calibration sets		Validation sets		RPD
		RMSEC	Rc	RMSEP	Rp	
Raw	PLS	0.352	0.8991	0.578	0.8079	2.12
SG		0.353	0.8983	0.592	0.7864	1.87
MSC		0.338	0.7072	0.563	0.6799	1.75
SNV		0.289	0.7086	0.521	0.6825	1.74
FD		0.276	0.9125	0.498	0.8215	2.21
SD		0.578	0.6058	0.623	0.8860	1.18
FD + SG		0.235	0.9447	0.286	0.9420	2.42
MSC + SG		0.340	0.9062	0.580	0.8543	2.17
MSC + FD		0.268	0.9430	0.167	0.8835	2.58
MSC + FD + SG		0.269	0.9424	0.167	0.8844	3.08
MSC + SD		0.695	0.6031	0.723	0.2932	1.61
MSC + SD + SG		0.678	0.7386	0.611	0.6620	1.51
SNV + SG		0.728	0.7249	0.627	0.7158	1.56
SNV + FD		0.285	0.8353	0.213	0.8131	1.74
SNV + FD + SG		0.728	0.8249	0.627	0.8158	2.54
SNV + SD		0.720	0.6463	0.731	0.6519	2.46
SNV + SD + SG		0.337	0.8082	0.124	0.9871	2.88
Raw	PCR	0.292	0.6872	0.304	0.7525	1.75
SG		0.387	0.8434	0.458	0.8248	1.65
MSC		0.463	0.7309	0.467	0.7095	1.58
SNV		0.439	0.7480	0.473	0.6569	1.39
FD		0.441	0.8478	0.452	0.8269	1.12
SD		0.563	0.8309	0.597	0.8195	1.24
FD + SG		0.250	0.8808	0.292	0.8509	2.35
MSC + SG		0.310	0.7836	0.391	0.6592	1.89
MSC + FD		0.340	0.8611	0.326	0.7250	2.11
MSC + FD + SG		0.306	0.6404	0.338	0.6711	2.23
MSC + SD		0.503	0.6437	0.682	0.6204	1.96
MSC + SD + SG		0.511	0.7681	0.613	0.7387	2.14
SNV + SG		0.558	0.5456	0.592	0.5730	2.39
SNV + FD		0.570	0.6772	0.576	0.6218	2.11
SNV + FD + SG		0.481	0.6398	0.526	0.6257	2.37
SNV + SD		0.495	0.5407	0.587	0.5119	2.05
SNV + SD + SG		0.392	0.7398	0.426	0.6857	1.87

### 3.4 Variable selection and models optimization

The initial dataset comprised an excessive number of spectral variables, many of which were irrelevant to the modeling process. To address this issue, various feature extraction methods were employed following preprocessing to eliminate redundant spectral information. This approach aimed to reduce the complexity of subsequent analyses and enhance the accuracy of the data interpretation. A comparative experimental analysis of Uninformative Variable Elimination Partial Least Squares Regression (UVE-PLSR), Competitive Adaptive Reweighted Sampling Partial Least Squares Regression (CARS-PLSR), and Successive Projections Algorithm Partial Least Squares Regression (SPA-PLSR) was conducted, with results summarized in Table 4.

**Table 4:** The modelling results from the different algorithm selection methods for NIR spectras

Algorithm	Variables	Models	Calibration sets		Validation sets		RPD
			*R*_C_	RMSEC	*R*_P_	RMSEP	
PLSR	Full-spectrum	—	0.9447	0.235	0.9420	0.286	2.42
UVE	442	PLSR	0.9831	0.1483	0.9611	0.1538	2.88
CARS	152	PLSR	0.9860	0.1340	0.9825	0.1270	3.27
SPA	266	PLSR	0.9675	0.1572	0.9556	0.1567	2.76

**Fig. 2:**
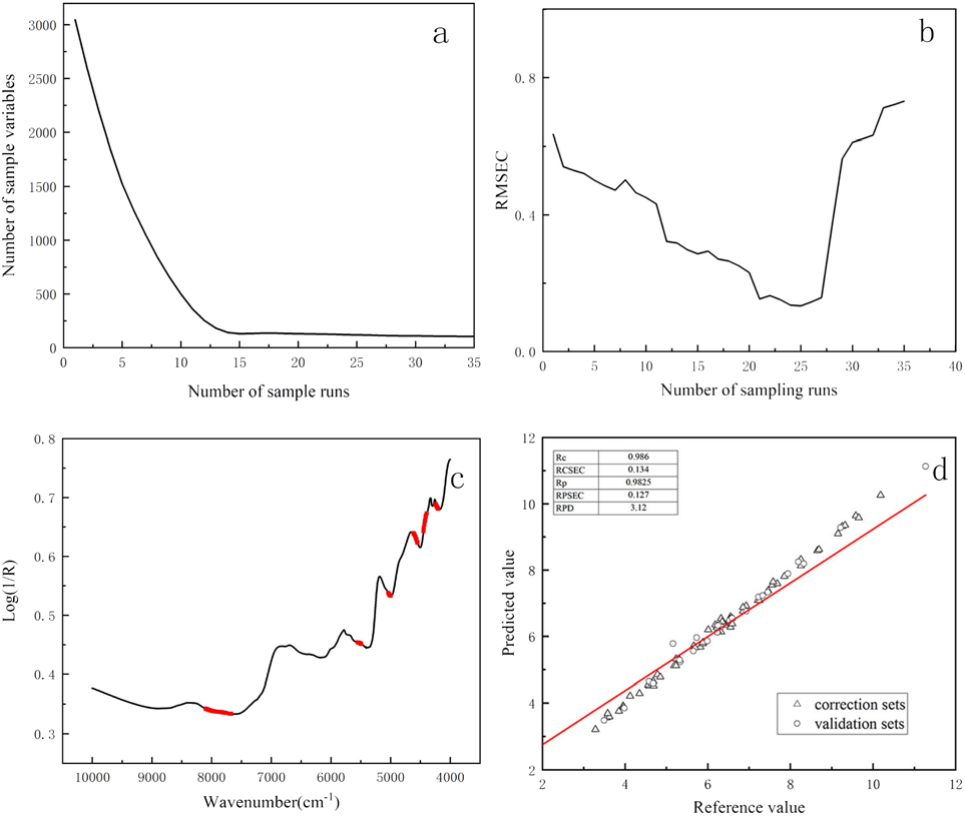
Optimization of spectral intervals and quantification models using CARS-PLS for TP/FAA in Wuyi Rock Tea. (a) The iterative variable selection process of the CARS algorithm; (b) the change in RMSECV with the number of iterations; (c) the feature wavelengths selected by CARS; and (d) the linear fitting plot of the quantification model.

Among the three feature wavelength selection algorithms (CARS, UVE, and SPA), each was found to effectively extract relevant features compared to full-spectrum analysis. The number of wavelengths retained after selection was 152 for CARS, 442 for UVE, and 226 for SPA, with the CARS-PLSR algorithm exhibiting the most favorable performance. The prediction model established using the CARS method demonstrated significant improvements over alternatives. The iterative selection process utilized by the CARS algorithm is illustrated in Fig. 2. As shown in Fig. 2-a, the exponential decline curve indicates a rapid initial decrease in the number of selected variables, which then stabilized as the number of iterations increased, thereby enhancing screening efficiency. In Fig. 2-b, the trend of the root mean square error of cross-validation (RMSECV) is depicted; it initially decreased before increasing again, achieving its minimum when the number of sampling iteration was 25. This pattern suggests that the algorithm effectively eliminated irrelevant spectral information, although further iterations risked excluding wavelengths correlated with tea polyphenols and free amino acids, which subsequently led to an increased in RMSECV.

For instance, in the case of the CARS, to understand the chemical basis of the model's predictive power, the distribution of these selected wavelengths was examined in relation to known absorption features of tea constituents (as outlined in Section 3.2 and Fig. 1). As illustrated in Fig. 2-c, the wavelengths selected by the CARS algorithm were predominantly concentrated within spectral regions corresponding to the characteristic functional groups of the target compounds. These regions are primarily associated with: (1) the combination vibrations of C–H and N–H bonds, which are fundamental to the structures of both polyphenols and amino acids; (2) the overtone and combination bands of C=O, C=C, and C=N bonds, characteristic of flavonoid-type polyphenols; and (3) the first-overtone vibrations of C–H bonds along with combination bands of C–C, C–O, and C–N bonds, reflecting the overall organic matrix. This alignment demonstrates that CARS successfully filtered out non-informative spectral noise while retaining variables corresponding to the fundamental vibrational modes of the chemical groups that determine the TP and FAA content. Therefore, the enhanced model performance achieved with CARS-PLSR (Table 4) can be attributed to its focused utilization of these chemically meaningful spectral regions for quantifying the TP/FAA ratio.

Through the analysis, a total of 152 wavelengths were ultimately identified as critical for predicting the phenol-to-amino acid ratio, following optimization, the model for Wuyi Rock Tea exhibited improved performance, with the *R*_c_ and *R*_p_ increasing from 0.9447 and 0.9420 to 0.9860 and 0.9825, respectively. Concurrently, the RMSEC and RMSEP decreased from 0.235 and 0.286 to 0.1340 and 0.1270. The RPD value also rose significantly from 2.42 to 3.27, as shown in Fig. 2-d, collectively indicating a substantial enhancement in the predictive accuracy of the CARS-PLS model. Moreover, the considerable reduction in the number of modeling variables led to a decreased optimal number of principal components, thereby simplifying the model structure and enhancing its stability. This approach not only preserved essential spectral features but also reduced computational complexity, ultimately optimizing the prediction model for the phenol-to-amino acid ratio in Wuyi Rock Tea.

### 3.5 External validation

After establishing the optimal prediction model for the TP/FAA ratio in Wuyi Rock Tea, we selected nine tea samples to evaluate the model's accuracy. The spectral data from these samples were input into the finalized prediction model, resulting in the calculated TP/FAA ratios. Each sample was subjected to duplicate measurements, with the average values recorded for analysis. For comparative purposes, the TP/FAA ratios of the same nine samples were also determined following the protocols outlined in ISO 14502-1-2005 and ISO19563-2017, with the average values from these determinations serving as reference data. The results obtained from both methodologies are summarized in Table 5.

**Table 5:** The results of external validation experiment

Test items	Sample number	Measured values	Predicted values	Arithmeticmean	Absolute difference	Relative deviation/%
TP/FAA	Sample1	5.72	5.22	5.47	0.25	4.57
	Sample2	4.82	4.66	4.74	0.16	3.38
	Sample3	6.55	6.92	6.74	0.37	5.49
	Sample4	4.72	4.87	4.80	0.15	5.36
	Sample5	6.85	7.14	7.0	0.29	4.14
	Sample6	4.81	4.92	4.86	0.11	3.85
	Sample7	8.26	8.01	8.14	0.25	3.44
	Sample8	7.56	7.22	7.39	0.34	4.60
	Sample9	4.98	5.42	5.20	0.47	8.46

As shown in the Table 4, the TP/FAA ratios determined using the standard method exhibited a close correspondence with those predicted by the NIR spectroscopy model, with absolute differences remaining within 10% of the arithmetic mean. This alignment not only confirms the reliability of the quantitative model but also highlights its potential to serve as a rapid and accurate alternative to traditional methods for assessing the phenol-to-amino acid ratio in Wuyi Rock Tea.

### 3.6 Analysis of the impact of moisture on the model

As with any NIR-based quantitative model, the predictive performance for the TP/FAA ratio could theoretically be influenced by variations in sample physical properties, among which moisture content is a critical factor.^40^ Water exhibits strong O–H absorption bands in the NIR region, and variations in moisture can induce baseline shifts, alter light scattering patterns, and introduce non-specific spectral variance that is not directly related to the target analytes.^41^ In this study, several proactive measures were implemented to mitigate this potential interference, as detailed in Section 2.1. Furthermore, the employed spectral preprocessing techniques, particularly derivative methods (FD, SD) and scatter correction (SNV), are effective in compensating for baseline and multiplicative effects often associated with minor physical variations. The high predictive accuracy and robustness of the final CARS-PLSR model (*R*_p_ = 0.9825, RPD = 3.27) under these controlled conditions indicate that the residual moisture-related variance within our sample set was effectively managed. The model's success demonstrates that, within the defined sample preparation and measurement protocol, the spectral signatures of polyphenols and amino acids for TP/FAA prediction can be reliably extracted despite the ubiquitous presence of water.

## 4. Conclusions

The robustness of various multivariable quantification methods, when integrated with near-infrared (NIR) spectroscopy, has been systematically analyzed for the efficient determination of TP/FAA ratio in Wuyi Rock Tea. Among the models developed in this study, the Partial Least Squares Regression with Competitive Adaptive Reweighted Sampling (PLSR-CARS), utilizing the FD + SD combination for data pretreatment, exhibited superior performance, characterized by enhanced accuracy and predictive capability. Both the correlation coefficients of calibration (*R*_c_) and prediction (*R*_p_) were observed to exceed 98%, while the ratio of Performance to Deviation (RPD) was greater than 3, indicating a robust correlation between the corrected TP/FAA data and the predictions generated by the NIR model. The reliability and accuracy of the established model were further corroborated through external validation experiments. The specific correlation coefficients were found to be *R*_c_ = 0.9860 and *R*_p_ = 0.9825, with the Root Mean Square Error of Calibration (RMSEC) and the Root Mean Square Error of Prediction (RMSEP) measured at 0.1340 and 0.1270, respectively, resulting in an RPD of 3.27. Compared to traditional methods for assessing the TP/FAA ratio in Wuyi Rock Tea, this novel method not only facilitates quicker response times but also address the limitations associated with more complex analytical procedures. Consequently, it is well-suited for rapid detection and real-time monitoring of the phenolic to amino acid ratio in Wuyi Rock Tea. This integration of NIR spectroscopy and chemometric techniques thus presents a significant advancement in the quantitative analysis of tea components.

It should be noted that the model established in this work was optimized specifically for Wuyi Rock Tea, whose unique biochemical profile and processing characteristics distinguish it from other tea varieties. Direct application of this model to other tea types (*e.g.*, green tea, black tea, or other oolongs) may lead to degraded prediction accuracy due to differences in chemical composition, particle size, moisture content, and spectral interference patterns. To extend this methodology to different tea matrices, future studies should consider the following: (1) recalibration using a representative set of samples from the target tea type; (2) adjustment of spectral preprocessing protocols to address matrix-specific scattering and noise; (3) re-evaluation of feature wavelength selection to capture tea-specific spectral signatures. Further research could also explore transfer learning or model fusion techniques to develop more universal calibration models capable of predicting TP/FAA ratios across multiple tea categories with minimal recalibration effort.

## Conflicts of interest

There are no conflicts to declare.

## Data Availability

Some or all data, models, or code generated or used during the study are available from the corresponding author by request.
